# Structured illumination microscopy for cancer identification in diagnostic breast biopsies

**DOI:** 10.1371/journal.pone.0302600

**Published:** 2024-05-09

**Authors:** Madeline Behr, Layla Alizadeh, Lyndsey Buckner-Baiamonte, Brett Roberts, Andrew B. Sholl, J. Quincy Brown

**Affiliations:** 1 Department of Biomedical Engineering, Tulane University, New Orleans, LA, United States of America; 2 Department of Pathology, Ochsner Medical Center, New Orleans, LA, United States of America; 3 Biorepository Unit, Ochsner Medical Center, New Orleans, LA, United States of America; 4 Department of Radiology, Ochsner Medical Center, New Orleans, LA, United States of America; 5 Department of Pathology, Touro Infirmary, New Orleans, LA, United States of America; University of Houston, UNITED STATES

## Abstract

Breast cancer is the second most common cancer diagnosed in women in the US with almost 280,000 new cases anticipated in 2023. Currently, on-site pathology for location guidance is not available during the collection of breast biopsies or during surgical intervention procedures. This shortcoming contributes to repeat biopsy and re-excision procedures, increasing the cost and patient discomfort during the cancer management process. Both procedures could benefit from on-site feedback, but current clinical on-site evaluation techniques are not commonly used on breast tissue because they are destructive and inaccurate. *Ex-vivo* microscopy is an emerging field aimed at creating histology-analogous images from non- or minimally-processed tissues, and is a promising tool for addressing this pain point in clinical cancer management. We investigated the ability structured illumination microscopy (SIM) to generate images from freshly-obtained breast tissues for structure identification and cancer identification at a speed compatible with potential on-site clinical implementation. We imaged 47 biopsies from patients undergoing a guided breast biopsy procedure using a customized SIM system and a dual-color fluorescent hematoxylin & eosin (H&E) analog. These biopsies had an average size of 0.92 cm^2^ (minimum 0.1, maximum 4.2) and had an average imaging time of 7:29 (minimum 0:22, maximum 37:44). After imaging, breast biopsies were submitted for standard histopathological processing and review. A board-certified pathologist returned a binary diagnostic accuracy of 96% when compared to diagnoses from gold-standard histology slides, and key tissue features including stroma, vessels, ducts, and lobules were identified from the resulting images.

## Introduction

Breast cancer is the second most common cancer diagnosis in women, after skin cancer, with almost 280,000 new cases predicted in the US in 2023 [1]. Suspicion of breast cancer is typically triggered by a new breast symptom or an abnormal annual mammogram, which is recommended by the American College of Radiology for all women 40 and older [2]. The results of a mammogram are stratified for abnormality according to the Breast Imaging Reporting and Data System (BI-RADS) and abnormal findings that cause suspicion of breast cancer are designated as a 4 or 5 on an ordinal scale of 0 (nondiagnostic) to 6 (confirmed cancer from biopsy). Patients with a BI-RADS score of 4 or 5 are counseled to undergo a diagnostic breast biopsy under ultrasound, 3D tomosynthesis (mammogram or stereotactic), or MRI guidance. The number of biopsies collected from a lesion of interest is case dependent, and varies by needle size, imaging modality, type of suspicious lesion, and presence of calcifications. Even with guidance, breast tissue heterogeneity, including density changes, fat content, and non-malignant cysts can impair the accuracy of a diagnostic biopsy procedure. Location inaccuracy during these procedures contributes directly to false negative results, which is about 2% for ultrasound guided biopsies and 9% for stereotactic biopsies [3]. Positive lumpectomy margins also occur at a high rate in clinical settings and frequently prompt re-excision procedures [4–6].

Currently, the assessment of breast biopsies and lumpectomy margins is conducted via histopathology, in which the samples are fixed in a formalin solution for up to 72 hours, before it is embedded in a paraffin block to create a formalin fixed paraffin embedded (FFPE) tissue block of the sample, which is the gold-standard for histology processing of tissues. Biopsies are then cut with a microtome, stained with hematoxylin and eosin (H&E) and mounted on a slide for pathologist assessment. In the fastest iteration of this process, histological processing can be achieved within a day of biopsy collection, but in clinical settings this process usually takes multiple days due to personnel availability and workload back up. This process is not compatible with any on-site assessment of breast biopsy samples at the point-of-care.

There are existing modalities for the rapid assessment of fresh tissues, known as rapid on-site evaluation (ROSE) techniques including frozen section analysis (FSA) and touch-prep cytology, but they are not commonly utilized in the clinic because they are destructive, provide inaccurate results, or require an on-site pathologist [7]. FSA is conducted by placing a fresh tissue sample on a freezing microtome which utilizes a dramatic temperature decrease to rapidly harden the sample which is then sectioned, stained and mounted on a slide for review [8, 9]. This procedure can be achieved within 20 minutes of biopsy acquisition [10], but is not widely adopted in breast cancer settings because the high fat content typical of this tissue amplifies the already present risk of nuclear aberrations and increases the rate of deferred diagnoses [11]. Additionally, it is difficult to distinguish abnormal nonmalignant structures in frozen breast tissue, especially if fibrosis or cysts are present [12]. The FSA process is inherently destructive to the tissue, which prevents subsequent standard histology processing of the same sample for confirmation or biomarker assessment and requires an on-site pathologist for slide reading. Touch-prep cytology does not suffer from the same destructive processing, as it is achieved by smearing the fresh sample on a slide, spraying the slide with fixative, and staining it according to the Diff-Quik technique [13]. This ROSE technique can be completed in a few minutes but suffers from a relatively low sensitivity (75%) and the accuracy of touch-prep cytology is further impeded by fat, fibrosis, and cysts common in breast tissue [13–15]. For touch-prep cytology to be a viable ROSE modality, a cytopathologist must be present at the point-of-care.

Since FSA and touch-prep cytology are not widely utilized in clinical breast cancer management, there is still a need for a reliable modality to assess breast tissue on-site. *Ex-vivo* microscopy is an emerging technology that utilizes optical-sectioning to generate histologic-analogous images of non- or minimally processed tissues at point of care and offers qualities that are promising for on-site tissue assessment in a clinical setting [16, 17]. Some of the most popular *ex-vivo* microscopy techniques include confocal microscopy [18–20], light sheet microscopy [21], microscopy with UV-excitation (MUSE) [22–24], multiphoton microscopy [25], nonlinear optical imaging (NLOI) [26], optical coherence tomography/imaging (OCT) [27], and structured illumination microscopy (SIM) [28–31]. Many of these optical-sectioning microscopies can be combined with fluorescence markers to label specific structures such as collagen, nucleic acids, and other biomarkers. Several *ex-vivo* microscopies have been combined with optical clearing methods to facilitate deeper imaging into samples [32–34]. These microscopy techniques have been demonstrated in the laboratory, but optical clearing steps are lengthy, which present a hinderance to clinical implementation, especially in the context of on-site feedback applications. As such, the most promising approaches for rapid clinical feedback leverage rapid 2D optical sectioning of fresh tissue surfaces, or 3D imaging that does not require optical clearing [35, 36]. Research in the field of *ex-vivo* microscopy continues to address these barriers, including the recent work by Conservano et. al., which recently presented a novel approach to confocal microscopy that can identify breast carcinomas within 10 minutes [37].

We have expanded the applications of SIM as a fast and non-destructive *ex-vivo* imaging technique for tissue samples. SIM utilizes a wide-field approach to optical sectioning which corresponds to faster surface imaging throughput when compared to beam-scanning approaches like confocal or multiphoton microscopy, or line-scanning approaches like OCT or light sheet microscopy. Usable imaging depth into uncleared tissue is limited to ~50 microns, thus and SIM maximizes efficiency by collecting images from uncleared tissue surfaces through an *en face* imaging approach rather than angled or line illumination. Compared to other *en face* wide-field optical sectioning strategies (e.g. MUSE, FF-OCT), SIM maintains the ability to use any visible spectrum excitation wavelength, and its optical sectioning performance and lateral and axial resolutions can be directly tuned by changing the illumination pattern frequency [38]. SIM has been successfully utilized to image biopsies from breast, prostate, and kidney [28–30, 39] and larger tissue samples including whole prostate resection surfaces [40] and partial nephrectomies [31]. Our lab has also investigated the use of inverted selective plane illumination microscopy (iSPIM) to generate 3D pseudo-H&E stacks from formalin fixed and optically cleared breast lumpectomy samples [41].

We propose SIM as a preferred *ex-vivo* microscopy technique for the on-site assessment of breast biopsies in a clinical setting because of its speed, contrast, and image quality. This is, to our knowledge, the first study of SIM imaging on fresh diagnostic breast biopsies. The non-destructive nature of sample staining and imaging for SIM preserves the sample integrity for subsequent processing of breast biopsies, which provides a key advantage as it allows for direct confirmation against gold-standard histology and any subsequent biomarker analysis. Preservation of sample integrity, rapid generation of a digital result for interpretation, and ease-of-use are features of SIM that make it attractive as a replacement for FSA or touch-prep cytology in clinical breast cancer management. In this study, we demonstrate that images generated via SIM from fresh diagnostic breast biopsies can be used to identify key structures and provide promising preliminary data for the assessment of cancer presence in these images at a speed compatible with potential on-site clinical applications. Additionally, we utilized a 10X and 20X imaging objective to analyze the image quality and imaging speed implications of higher objective lens magnification and resolution on SIM imaging of breast biopsy samples. The findings from this study position SIM as a promising modality for on-site rapid breast tissue characterization, in the setting of diagnostic biopsy or breast tumor margin assessment.

## Materials and methods

### Instrumentation

**Fig 1:**
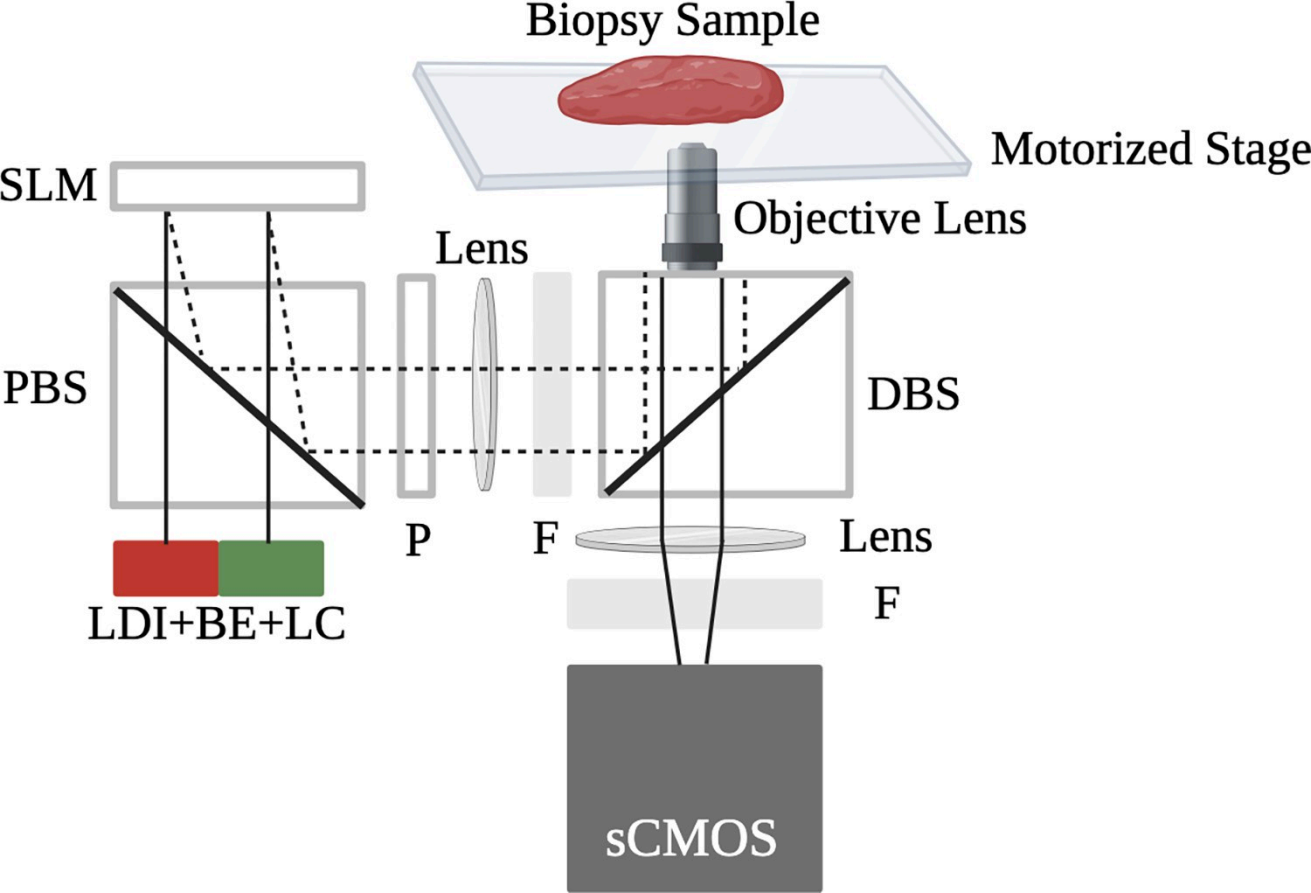
A schematic of the SIM instrumentation used to image samples in this study. Explanations of abbreviations used in this schematic are: BE- beam expander, LC- light collimator, PBS- polarized beam splitter, SLM- spatial light modulator, P- polarizer, F- filter, and DBS- dichroic beam splitter. This figure was created with BioRender.com.

The samples for this study were imaged using a customized, multicolor SIM system which utilized a pattern projection unit based on a liquid crystal on silicon (LCoS) spatial light modulator (3DM, Fourth Dimension Displays) connected to an automated epifluorescence microscopy platform (RAMM, Applied Scientific Instrumentation) by 30 mm cage system components (Thorlabs). A multiline laser engine (LDI 6, Chroma) provided illumination and excitation and emission wavelengths were discriminated using custom manufactured multiband optical fibers and beamsplitters (Chroma). Optical sectioning using incoherent SIM was performed using the square-law demodulation algorithm previously described by Neil and colleagues [42]. Both 10X (Nikon Plan Apo 10X 0.45 NA) and 20X (Nikon Plan Apo 20X 0.75 NA) objective lenses were used for imaging. This SIM system has a 1.3 mm x 1.3 mm single-frame FOV with 1.3 μm resolution (10X objective lens) or 660 μm x 660 μm single-frame FOV with 0.64 μm resolution (20X objective lens), with resolution Nyquist-limited by the camera pixel size in both cases. A schematic of the SIM system used for data collection is presented in Fig 1.

### Sample collection

All samples for this study were collected under the protocol approved by the Ochsner Health System Institutional Review Board and the Tulane University Biomedical Institutional Review Board and in accordance with all approved procedures (protocol code #2018.314.ROSE). This study had a recruitment period of 12/5/2018 to 11/4/2021. Eligible patients who were scheduled for either a mammogram or ultrasound-guided breast biopsy were approached for participation by research staff and written informed consent was obtained. A single core biopsy, for research purposes, with either 3D tomosynthesis or ultrasound guidance was collected from each participant during the procedure. Research biopsy specimens were placed in pre-prepared and labelled collection vials of 7.4 pH PBS immediately after collection by the radiologist and the vials were packed on ice until transportation to the lab within 2 hours of excision. The average age of the patients consented for this study was 54 years old (19–82 years). 27 of the biopsies were collected with ultrasound-guidance and 20 were with 3D tomosynthesis. An initial sample subset was used for optimization of staining concentration and time, sample handling and mounting methods, and imaging protocols with the fresh breast biopsy tissues. These samples, as well as those that suffered from an autofocus mechanism failure or camera oversaturation, were excluded from the final data set. All in-focus and non-saturated images were retained for pathologic review. There were no patient-related adverse events associated with the additional core sample acquisition.

### Staining

Breast biopsies were stained with fluorescent dyes DRAQ5 (Biostatus, LTD) and Alcoholic Eosin-Y 515 (Leica) to replicate the standard histological stains of hematoxylin and eosin [29]. The samples were individually submerged in 200 μL of a 50 mM solution of DRAQ5 in PBS (Fischer Scientific) for three minutes, then immediately submerged in 1 mL of PBS to rinse the excess stain. The biopsy was then submerged in 200 μL of a 50% w/v solution of Alcoholic Eosin-Y 515 in ethanol (Leica) for 10 seconds. Finally, the sample was dipped in 1 mL of 1% Surgipath acid Alcohol (Leica) to remove the excess Eosin-Y 515 stain and blotted on a fresh KimWipe (Kimberly-Clark) to absorb the excess liquid from the biopsy. The stained and lightly dried biopsy was mounted between two 0.15 mm D x 24 mm W x 50 mm L coverglass slides with gentle pressure for imaging.

### Imaging

Fluorescence images of each breast biopsy sample were taken using a custom software automated microscope. The system’s motorized controls of the sample stage (X-Y-plane) and the objective lens (Z-plane) were used to determine the clearest initial focus position at the tissue surface as well as the imaging dimensions required to image the sample surface through the live view. Additionally, the live feed was used to optimize the power of the 640 nm laser and the 528 nm laser. Stage and objective lens movements necessary to image the designated sample area in a serpentine pattern were handled automatically by the imaging software. A custom autofocus routine was used to address variations in tissue height caused by the density heterogeneity of breast biopsy samples to create a more consistent imaging plane. The autofocus parameters consisted of re-focusing the objective lens every 4 scan positions in the mosaic via a hill-climbing method to optimize the contrast of only the 640 nm (DRAQ5) channel with a step size of 4 μm in the Z-direction. The three patterned images per frame for each excitation channel were combined to create a single optically-sectioned image per frame per excitation channel [42]. The resulting SIM images were stitched together using Fiji [43] to create two gray-scale images of the imaging surface: one containing information from the DRAQ5 channel and the other containing information from the Eosin-Y channel. The two gray-scale images were combined to create one psuedocolored image through the method of by Giacomelli et. al. [44], adapted for SIM application by Elfer et. al. [45] and as demonstrated in our prior publications [29, 46]. In this method, pseudocoloring parameters are chosen by research personnel to define the intensity and gamma values used to weight the coloring of each gray-scale image. A frame with strong features in both channels is imported into a customized MATLAB script where these values are adjusted and the effect on the chosen frame is displayed in real time. Once a combination of parameters that supports feature visualization is identified, the same MATLAB script applies the pseudocoloring equations to generate a “pseudo-H&E” image of the entire biopsy.

### Histopathology

Upon completion of sample imaging, breast biopsies were mounted in a histology cassette and placed in a 10% buffered formalin solution for a minimum of 6 hours and then sent to histology for processing. This processing consisted of cutting three sections 4 μm apart in the Z direction from the biopsy which was then stained with hematoxylin and eosin.

### Blinded pathologist review

A board-certified pathologist was provided sets of pseudo-H&E SIM images and the corresponding histology slides in sets of approximately 12 (ranging from 8 to 15) and blinded to their correlation. The pathologist did not receive any training specific to interpreting SIM images prior to evaluating the images for this study. HistomicsUI (Kitware, Inc.), an open-source web-based pathology image viewer was used by the pathologist to visualize and annotate the generated SIM images. The pathologist reported their impression of the sample as malignant vs. nonmalignant. Subsequent histology slides were also reviewed via standard clinical microscope procedures and the binary diagnosis, malignancy type (if applicable) and additional commentary were reported. An additional pathologist rater who is experienced with SIM provided interpretation of SIM images to provide additional tissue structure identification and to aid in interpretation of accuracy results. Pathology reports from the hospital regarding the overall diagnosis for the patient were collected as reference.

## Results

The specifications of the patient population included in this study are presented in Table 1. Patients were required to be over 18, not a member of a vulnerable population (incarcerated, cognitively impaired, etc.), and undergoing their biopsy procedure with a radiologist listed on the IRB to be eligible to participate in this research project. All patients involved in this study are female. The preoperative diagnoses for the patients in this study contain 19 suspicious masses, 12 calcifications, and 16 other causes for biopsies, including an abnormal mammogram and fibrocystic changes. Of the patients with masses, 14 were benign and 5 were malignant, for those with calcifications, 9 were benign and 3 were malignant, and finally the other causes had 11 benign and 5 malignant results.

**Table 1:** Summary of patient demographics for samples collected for this study including age, biopsy guidance method, side of body, and suspicion of lymph node involvement. An expanded table of patient details for each sample is provided in S1 Table.

Age (years)		Biopsy Guidance (n)		Side of Body (n)		Suspicion of Lymph Involvement? (n)	
Mean	54	Ultrasound	27	Left	25	Yes	2
Max.	82	Mammogram	20	Right	16	No	44
Min.	19			Bilateral	5	N/A	1
St. Dev.	13			N/A	1		

**Fig 2:**
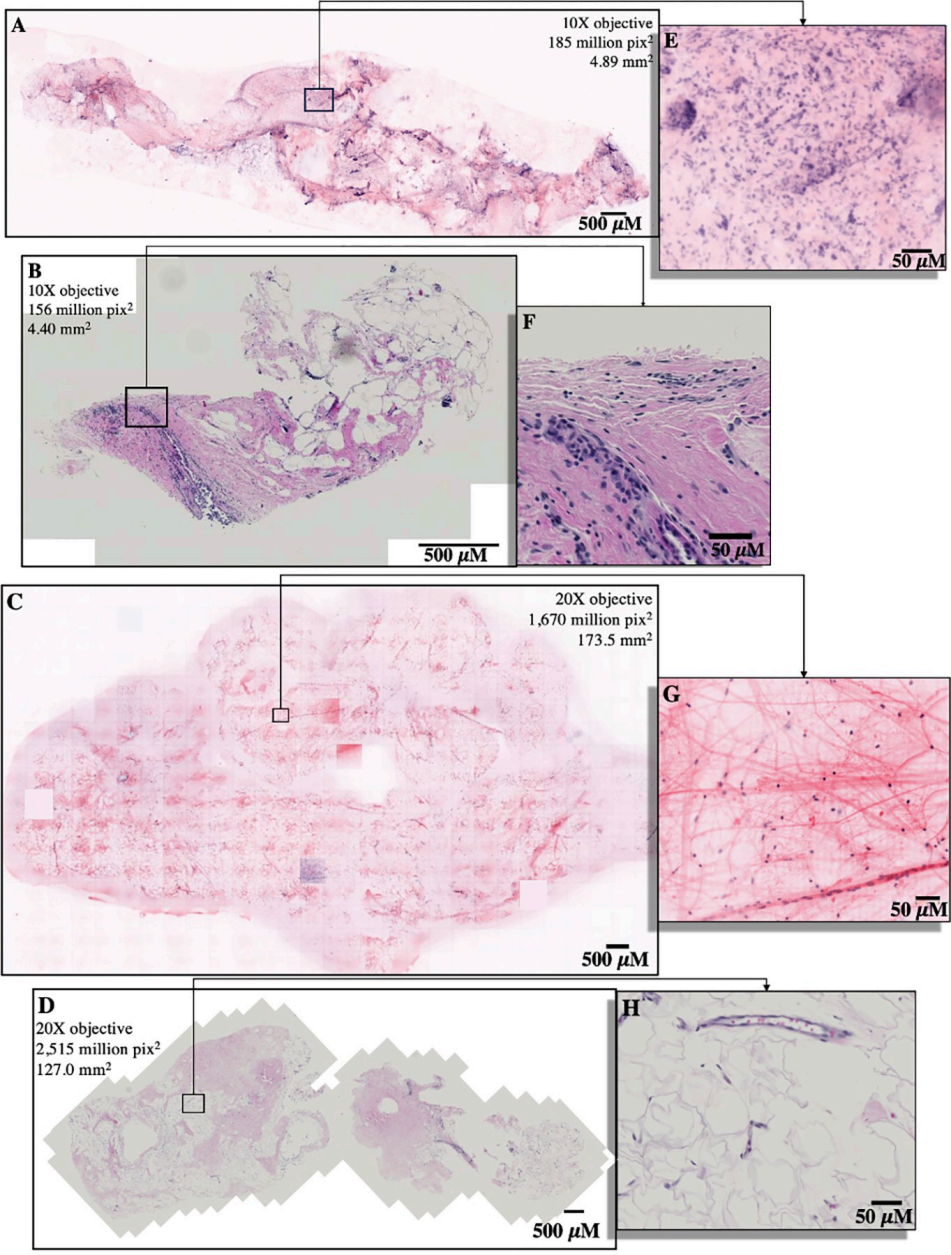
An ultrasound guided breast biopsy with invasive ductal carcinoma imaged with a 10X objective on SIM (**A**), and the subsequent histology slide from the biopsy (**B**). A stereotactic breast biopsy imaged with a 20X objective on SIM (**C**), and the subsequent histology slide from the biopsy (**D**). A zoomed in selection demonstrates the large nuclei of unstructured invasive ductal carcinoma cells found in both SIM images and histology slides (**E, F**). The ant-like morphology of healthy breast stroma can be seen in selected zooms on SIM images and histology slides from breast biopsies (**G, H**).

Optically-sectioned SIM images collected from fresh breast biopsies offered a larger tissue area per section than their histology slide counterparts when either the 10X or 20X objective lens was used. This is primarily due to tissue shrinkage while creating the FFPE block as well as inherent tissue loss that is associated with the processing steps required create histology slides. Fig 2 shows macro-scale SIM images taken with a 10X and 20X objective lens respectively, and the subsequent histology slides. Histology slides were scanned using a Zeiss AxioScan Z.1 Slide Scanner (Zeiss).

**Fig 3:**
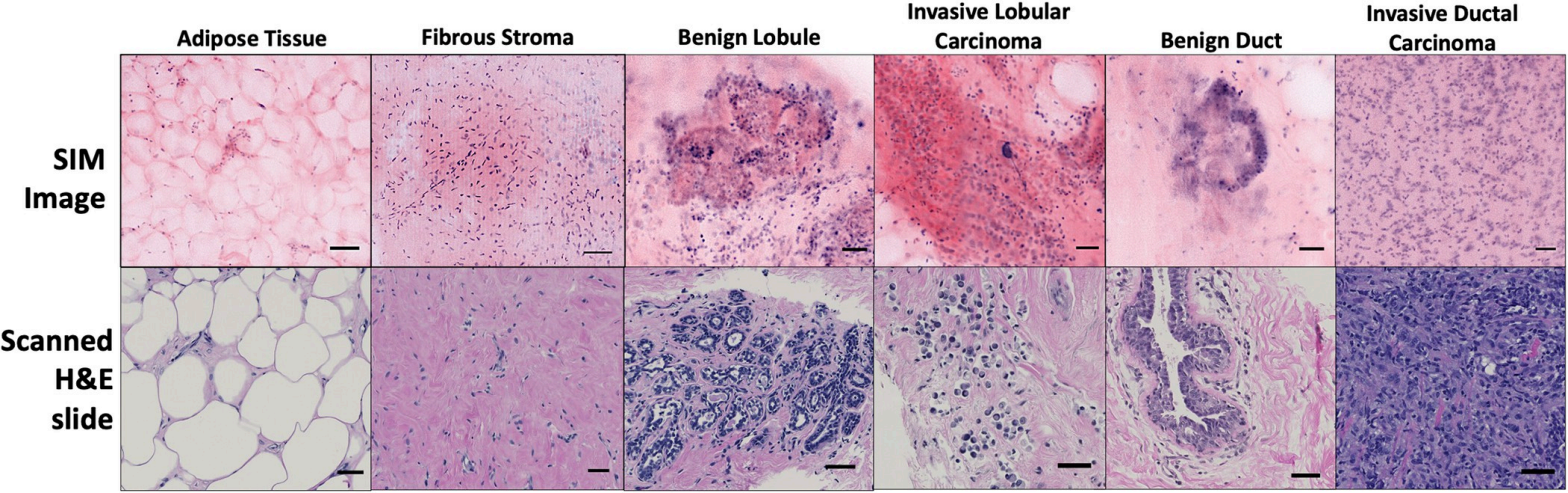
High resolution images of common breast structures from whole SIM images of breast biopsies and subsequent histology slides. Scale bars represent 50 μm.

Although SIM images of fresh breast biopsies are large, consisting of hundreds to thousands of millions of pixels, this microscopy technique still maintains high resolution. This allows for the identification of relatively small structures in large images. The pathology image viewing platform (HistomicsUI) facilitated the pathologist to zoom into the biopsy images up to 40X to view the image at multiple resolutions. This feature was highly utilized for the assessment of cancer presence and used to annotate key structures. Examples of healthy and malignant breast morphology as seen in SIM images and on histology slides are presented in Fig 3.

The pathologist returned a binary diagnosis from the digital pseudo-H&E images created by SIM from fresh, diagnostic breast biopsies as well as from the subsequent histology slides that were generated by gold-standard histological processing. The pathologist was blinded to their correlation and the diagnoses were retrospectively compared by research personnel. The confusion matrix and statistics of the pathologist’s diagnoses from 47 SIM images compared to gold standard H&E slides are presented in Table 2.

**Table 2:** Confusion matrix for the pathologist rater and diagnostic results summary of SIM images against gold-standard histopathology slides. Positive predictive value is abbreviated PPV and negative predictive value is abbreviated NPV in this table.

				H&E Diagnosis		
				Benign	Malignant	
				35	12	
	SIM Diagnosis	Benign	35	34	1	
		Malignant	12	1	11	
**True Positives**	**True Negatives**	**Sensitivity**	**Specificity**	**PPV**	**NPV**	**Accuracy**
11	34	92%	97%	92%	97%	96%

The biopsy sizes in this study ranged greatly as a product of needle size- 14 gauge for ultrasound guided procedures and 8 gauge for stereotactic ones. This contributed to a wide range in imaging times. The samples imaged with the 10X objective lens were imaged in an average time of 4:21 (range: 0:22–19:18, st. dev.: 4:50) and those imaged with the 20X objective lens were imaged in an average time of 10:36 (range: 1:20–37:44, st. dev.: 9:22). These imaging times correspond to an average image size of 0.91 cm^2^ (range: 0.1–4.2 cm^2^, st. dev.: 0.25 cm^2^) for the 10X objective sample set and 0.92 cm^2^ (range: 0.06–2.9 cm^2^, st. dev.: 0.14 cm^2^) for the 20X objective sample set. The autofocus mechanism was utilized to improve the final image for 3 of the samples imaged with a 10X objective and 9 of the samples imaged with the 20X objective. Within the data set of the samples imaged with a 10X objective, the samples with autofocus had imaging times ranging from 12:14–19:18 and the autofocus imaging times ranged from 18:29–37:44 for samples imaged with a 20X objective. After image acquisition, processing of SIM images was achieved within 40 seconds and the pathologist reported reading each image in 60 to 90 seconds. In total, from the collection of fresh breast tissue to delivering a malignancy determination, the process took an average of 7.85 minutes when samples were imaged with the 10X imaging objective and 14.1 minutes when the 20X imaging objective was used. This compares very favorably to results reported in a large study on FSA, where processing and interpretation times were 14.4 minutes and 8.2 minutes respectively [47].

## Discussion

In this work we conducted a preliminary assessment of the ability to identify cancer presence in fresh diagnostic breast biopsies through pseudo-H&E SIM images. The breast biopsies were stained and imaged within 2 hours of removal, unlike our prior study involving breast tissue, which was conducted on banked specimens [29]. When compared to other tissues that are diagnosed via biopsy like kidney, lung, and prostate, breast tissue has a large range of nonmalignant structures and variations that are problematic for current and emerging technologies. Relatively high fat content, cysts, and fibrosis can cause sharp changes in tissue density over small distances, which dramatically shifts the optical contrast and can impede consistent visualization of breast tissue [48]. This same heterogeneity hinders the application of FSA or touch-prep cytology as ROSE techniques. Between breast biopsy sampling location faults and inadequate ROSE methodologies, the need for a novel way to discern cancer presence in breast biopsies on-site is apparent. In this investigation we found promising preliminary results that SIM can overcome the optical challenges associated with breast tissue, be used to identify key structures, and even distinguish between malignant and nonmalignant samples with a high success rate when compared to its gold-standard histology counterpart. Optimal structure identification in SIM images is crucial for further applications of this technology towards clinical implementation. In this work, pathologists were able to identify characteristics of key breast morphology in SIM images including the cell clusters from benign lobes, the large and unorganized nuclei of malignant lobes, and the sheets of unstructured cells indicative of invasive ductal carcinoma. Well documented examples of common breast structures in SIM images are crucial to obtaining confident and correct diagnoses in future works. This need is highlighted in the false positive case in which the presence of fibrocystic changes mimics the sharp gland structure of carcinoma morphology and led to an incorrect malignant read from the SIM image. In the data set of 47 biopsies presented to the pathologist, a 92% sensitivity, 97% specificity, and 96% accuracy was achieved when binary diagnoses from SIM images were compared to diagnoses obtained from subsequent histology slides. Even without prior training on SIM images, the diagnosing pathologist returned promising preliminary data that encourages continued investigation.

As a component of this study, we investigated 10X and 20X objectives as imaging lenses for SIM imaging of fresh breast biopsies. The 10X objective was advantageous because it offers a thicker imaging plane, which is helpful to retain more nucleic information in fattier areas of breast biopsies. However, the images are lower resolution when compared to those from the 20X objective. Imaging the breast biopsies with the 20X objective offered higher resolution but at the tradeoff of an increased risk of camera oversaturation, specifically from the 528nm excitation of Eosin-Y, due to the higher light-gathering ability of that objective lens, and the minimum achievable intensity of the illumination laser. Additionally, the thinner imaging plane of the 20X 0.75NA objective lens made achieving proper focus on the fresh tissue surface more challenging. The focal challenges associated with 20X objective lens contributed to the false negative case when the high fat content combined with wet tissue impacted the contact of the tissue to the slide and impeded the proper visualization of epithelial cells and stroma relationship necessary to identify malignancy. In this preliminary investigation, we observed that while the 20X 0.75NA objective lens offers more detailed nuclear features, the improvement over the 10X 0.45NA was not enough to impact evaluation of malignancy and came at the expense of decreased speed and more challenging imaging considerations (including substantially narrower depth-of-focus and increased optical throughput which could overwhelm the camera sensor). Further research is necessary to address the imaging challenges associated with a thinner imaging plane and high light collection efficiency from the 20X objective lens to harness the higher resolution imaging to fresh breast tissue.

Beyond image quality and diagnostic accuracy, the time required for image generation is a crucial component of any on-site microscopy technique. In this study, we demonstrated that the total time required to create a pseudo-H&E image from a fresh diagnostic biopsy is compatible with on-site implementation. Staining and mounting the fresh biopsy on the imaging system requires about 5 minutes and the total processing time after imaging can be completed within 7 minutes. Imaging time of the freshly excised breast biopsy is dependent on the size of the biopsy and magnification of the objective lens. The average imaging time for biopsies imaged with a 10X objective (4:21) and a 20X objective (10:36) are both comparable to the speeds required for FSA [10]. Imaging with the 20X objective requires more time per biopsy because more frames are required to image the same area. Additionally, autofocus was employed more frequently during imaging with the 20X objective which increased the total imaging duration. The entire protocol from excision to image generation can be achieved in approximately 10 minutes for SIM images collected using a 10X objective and approximately 20 minutes for SIM images collected using a 20X objective. The demonstrated speed of the SIM staining, imaging, and processing protocol is comparable with on-site clinical implementation, and could give radiologists additional information at point-of-care to educate procedural decisions.

The binary diagnostic accuracy and speed of image generation from fresh diagnostic breast biopsies through SIM as demonstrated in this preliminary study has the potential for dramatic implications in a clinical setting. As a potential ROSE modality, SIM offers two distinct advantages when compared to FSA and touch-prep cytology: the generation of a digital image does not require an on-site pathologist for slide assessment, and the nondestructive nature of biopsy processing allows for histopathological confirmation or downstream biomarker analysis. The direct-to-digital pseudo-H&E image generated via SIM creates a workflow that supports remote pathologist feedback via the cloud, and could specifically serve rural communities that may not have an on-site pathologist to perform rapid on-site evaluation procedures. Beyond the biopsy procedure, SIM could be used to address the rate of positive margins seen in lumpectomies and mastectomies as surgical interventions for breast cancer [49]. In this future application, clinicians could utilize SIM as a tool to assess surgical margins in the operating room to identify positive margins on-site instead of waiting for the full histopathological work-up currently required. Investigation into this use would involve the combination of research our lab has done on partial nephrectomies [31] and radical prostatectomies [40] with the preliminary data and protocols presented in this work.

Future works involving the application of SIM as an *ex-vivo* microscopy technique to assess fresh breast biopsies will expand on this preliminary investigation. While the data in this study is promising, a study including a larger data set and additional pathologists is necessary to continue to validate the utility of SIM in an on-site clinical setting. This study is limited to investigating SIM as a tool for rapid on-stie evaluation of presence of malignancy, as a direct replacement for FSA or touch-prep cytology, rather than for primary diagnosis. The value of SIM as a primary diagnosis method to replace gold-standard permanent H&E pathology could be tested directly in a future study. However, since permanent FFPE histology is relatively inexpensive and entrenched technology and it currently serves as the definitive gold standard, there would need to be a demonstration of accuracy improvement over standard histopathology to justify its adoption. This is in contrast with on-site assessments, such as the detection of malignancy in biopsies or at excised tumor margins, where the value of a rapid assessment is to educate the completeness of the biopsy or tumor removal procedure in real-time, not provide the definitive diagnosis. We did not seek to determine the utility of fresh-tissue SIM as a replacement for primary diagnosis from FFPE histopathology. Further studies could provide meaningful insight about SIM’s potential as a stand-alone primary diagnostic modality. Additionally, through this study we identified technical limitations of SIM, particularly with the 20X objective lens, stemming from the optical heterogeneity of breast tissue. The implementation of a reliable autofocus mechanism could address this challenge by automatically adjusting the imaging plane, ensuring optimal focus for each imaging frame. Additional investigation is necessary to minimize the risk of camera oversaturation by Eosin-Y, which contributed to a loss of information in sections of a few biopsies. This work presents promising preliminary findings for the application of SIM as an *ex-vivo* microscopy modality for the on-site assessment of breast biopsy tissue. High binary diagnostic accuracy and fast image generation indicate that SIM could provide meaningful on-site feedback in clinical settings, and possibly address the lack of ROSE modalities for breast tissues.

## Supporting information

S1 Table Patient demographic for collected study samples including age, biopsy guidance method, side of body, mammogram-based diagnosis, and suspicion of lymph node involvement. ^±^This patient had a prior mastectomy; the study biopsy was collected from a new mass in reconstructed breast tissue. *Patient data was not available for sample OB0037.
(DOCX)
