# Supplementary material for: Structured illumination microscopy for cancer identification in diagnostic breast biopsies
Source: PLoS One. 2024 May 9;19(5):e0302600. doi: 10.1371/journal.pone.0302600 (PMC11081287; doi:10.1371/journal.pone.0302600)
Supplement: S1 Table — ±This patient had a prior mastectomy; the study biopsy was collected from a new mass in reconstructed breast tissue. *Patient data was not available for sample OB0037. (DOCX) [file pone.0302600.s001.docx]

| **Sample ID** | **Age** | **Biopsy Guidance** | **Location** | **Pre-Operative Diagnosis** | **Suspicion of Lymph Involvement?** |
| --- | --- | --- | --- | --- | --- |
| **OB001** | 35 | Ultrasound | Left | Possibly intraductal mass | No |
| **OB002** | 59 | Ultrasound | Left | Mass | No |
| **OB003** | 54 | Mammogram | Right | Calcifications | No |
| **OB004** | 42 | Ultrasound | Left | Mass | No |
| **OB005** | 19 | Ultrasound | Right | Mass | No |
| **OB006** | 55 | Mammogram | Right | Calcifications | No |
| **OB007** | 60 | Mammogram | Right | Calcifications | No |
| **OB008** | 40 | Ultrasound | Right | N/A | No |
| **OB009** | 46 | Ultrasound | Left | Mass | No |
| **OB010** | 59 | Ultrasound | Left | Mass | No |
| **OB011** | 59 | Ultrasound | Left | Mass | No |
| **OB012** | 82 | Mammogram | Left | Calcifications | No |
| **OB013** | 68 | Ultrasound | Left | Mass | No |
| **OB014** | 49 | Mammogram | Right | Calcifications | No |
| **OB015** | 38 | Mammogram | Left | Architectural distortion | No |
| **OB016** | 41 | Ultrasound | Bilateral |  | Yes |
| **OB017** | 35 | Ultrasound | Left | Mass | No |
| **OB018** | 81 | Ultrasound | Left | Mass | No |
| **OB019** | 66 | Mammogram | Left | Calcifications | No |
| **OB020** | 77 | Ultrasound | Left | N/A | No |
| **OB021** | 65 | Mammogram | Left | Asymmetry | No |
| **OB022** | 66 | Ultrasound | Left | Fibrocystic changes | No |
| **OB023** | 62 | Mammogram | Left | Calcifications | No |
| **OB024** | 55 | Ultrasound | Right | Intraductal Mass | No |
| **OB025** | 66 | Mammogram | Left | Calcifications | No |
| **OB026** | 63 | Ultrasound | Both | Mass | Yes |
| **OB027** | 65 | Mammogram | Right | Asymmetry | No |
| **OB028** | 69 | Mammogram | Right | Calcifications | No |
| **OB029** | 40 | Ultrasound | Right | Mass | No |
| **OB030** | 65 | Mammogram | Right | Mass | No |
| **OB031** | 44 | Ultrasound | Left | Mass | No |
| **OB032** | 62 | Ultrasound | Left | Focal change of echogenicity | No |
| **OB033** | 49 | Ultrasound | Right | Mass | No |
| **OB034** | 37 | Ultrasound | Left | Fibrocystic changes | No |
| **OB035*** | 46 | Ultrasound | Right | Mass | No |
| **OB036** | 48 | Ultrasound | Right | Focal asymmetry | No |
| **OB037** | N/A | Ultrasound | N/A | N/A | N/A |
| **OB038** | 44 | Ultrasound | Left | Mass | No |
| **OB039** | 59 | Ultrasound | Right | Abnormal mammogram | No |
| **OB040** | 51 | Mammogram | Left | Calcifications | No |
| **OB041** | 60 | Mammogram | Bilateral | Architectural distortion | No |
| **OB042** | 49 | Mammogram | Left | N/A | No |
| **OB043** | 60 | Mammogram | Right | Calcifications | No |
| **OB044** | 46 | Mammogram | Left | Calcifications | No |
| **OB045** | 36 | Ultrasound | Left | Mass | No |
| **OB046** | 58 | Mammogram | Bilateral | Abnormal mammogram | No |
| **OB047** | 58 | Mammogram | Bilateral | Abnormal mammogram | No |
